# Short-Term Effect of Ambient Temperature and the Risk of Stroke: A Systematic Review and Meta-Analysis

**DOI:** 10.3390/ijerph120809068

**Published:** 2015-07-31

**Authors:** Hui Lian, Yanping Ruan, Ruijuan Liang, Xiaole Liu, Zhongjie Fan

**Affiliations:** Department of Cardiology, Peking Union Medical College Hospital, Peking Union Medical College, Chinese Academy of Medical Sciences, Beijing 100730, China; E-Mails: lianhui1988@163.com (H.L.); yanping.ruan@163.com (Y.R.); 18911122174@163.com (R.L.); lxlryyc@sina.com (X.L.)

**Keywords:** stroke, temperature change, short-term, meta-analysis

## Abstract

Background and Purpose: The relationship between stroke and short-term temperature changes remains controversial. Therefore, we conducted a systematic review and meta-analysis to investigate the association between stroke and both high and low temperatures, and health assessment. Methods: We searched PubMed, Embase, Cochrane, China National Knowledge Infrastructure (CNKI) and Wanfang Data up to 14 September 2014. Study selection, quality assessment, and author-contractions were steps before data extraction. We converted all estimates effects into relative risk (RR) per 1 °C increase/decrease in temperature from 75th to 99th or 25th to 1st percentiles, then conducted meta-analyses to combine the ultimate RRs, and assessed health impact among the population. Results: 20 articles were included in the final analysis. The overall analysis showed a positive relationship between 1 °C change and the occurrence of major adverse cerebrovascular events (MACBE), 1.1% (95% confidence intervals (CI), 0.6 to 1.7) and 1.2% (95% CI, 0.8 to 1.6) increase for hot and cold effects separately. The same trends can be found in both effects of mortality and the cold effect for morbidity. Hot temperature acted as a protective factor of hemorrhage stroke (HS), −1.9% (95% CI, −2.8 to −0.9), however, it acted as a risk factor for ischemic stroke (IS), 1.2% (95% CI, 0.7 to 1.8). Conclusion: Short-term changes of both low and high temperature had statistically significant impacts on MACBE.

## 1. Introduction

The relationship between global climate changes and their effects on human health have been taken into the hub of public attention [1,2]. Among the numerous climate factors, the biggest concern focused on the impact of temperature. Previous studies concentrated on the association between both low and high ambient temperature and mortality [3,4,5,6], and a nonlinear U-, V- or J-shaped relationship has been reported [7,8,9], which suggested that in a moderate range of temperature (usually 25th to 75th percentiles of temperature in the whole year in a certain place), the mortality rate stayed low; below or above which, the health outcome increased. Cardiovascular diseases are among the hot spots. We usually found a positive relative risk (RR) for stroke in the subgroup analyses among those reports.

Carried out in 2010, the Global Burden of Diseases (GBD 2010) ranked stroke as the second most common cause for mortality [10]. However, less attention has been paid to the health effect of stroke and climate. The main outcomes of the studies were mortality and morbidity, and subtypes of stroke can be classified into two main types: Ischemic Stroke (IS) and Hemorrhagic Stroke (HS). Some of them found a U- or V-shaped relation, as expressed above. However, not all of the studies discovered the positive relationship. Therefore, we conducted a systematic meta-analysis to investigate the association between temperature and the risk of stroke.

## 2. Methods and Materials

### 2.1. Search Strategy and Study Criteria

We performed a systematic literature search of PubMed, Embase, Cochrane, CNKI and Wanfang Data up to the date 14 September 2014. Key words related to exposure (air temperature) combined to endpoint-related words (stroke, cerebrovascular disease) were used. All searches used the mesh methods to ensure accuracy. For example, the search strategy for PubMed was (“Cerebrovascular Disorders” [Mesh]) AND “Temperature” [Mesh]). In addition, we consider related references found in our literature search in order to cover all the studies.

All original time-series or case-crossover studies that presented original data about stroke—morbidity or mortality or any subtype—in response to exposure to ambient temperature were included. As some studies showed that the magnitude of temperature on the outcome did not vary with the exposure measure, we included all temperature measurements [11,12,13]. We excluded commentaries, summaries, reviews, case reports, case series, editorials, animal studies, toxicological studies, duplicates, and studies that reported another association. Because the main aim of the study is to find the relationship between change of temperature and health effects, we also excluded articles focused on the long-term effects. We ruled out studies only showed non-linear temperature–effect curves without the 95% confidence intervals (95% CI), which are necessary in meta-analyses. In cases of missing data (those we cannot extract), the authors were contracted for additional information. If no answer was obtained, the study was excluded.

### 2.2. Study Selection

The results of all titles and abstracts were merged into the Endnote, and duplicates were removed. All abstracts and titles were screened by two independent reviewers (Hui Lian and Yanping Ruan) to delete the irrelevant citations. Full-text articles of the potential eligible studies that met selection criteria were downloaded and read for inclusion in the systematic review. The reviewers then compared the results. If different opinions appeared, the first step was discussing. When we could not reach agreement after discussion, arbitration was sought from a third reviewer (Zhongjie Fan).

### 2.3. Quality Assessment

According to the Cochrane Handbook [14], and related literatures and articles [15,16], we evaluated the study quality with consideration of the following aspects: study design, study populations, sample size, statistical methods, measures of air temperature, adjusted confounders including meteorological factors, air pollutants, long-term trends, DOW, and technique of stroke diagnoses.

### 2.4. Data Extraction and Publication Bias

The data extraction was conducted independently by authors (Hui Lian and Yanping Ruan). A standardized checklist was used to extract data from studies that met the inclusion criteria. Data collected were: a full description of studies including title, author and year of publication, outcome investigated published journal, study design, location and period, statistical analysis model, number of events, variables controlled for, lag patterns and effect estimate. Effect estimate had to be presented as a Poisson or negative binomial regression coefficient, percent change, RR, or odds ratio (OR). As some of the studies included a series of cities and areas separately and pooled the estimates to reach the results, we separately extract the effect estimates for each city instead of the pooled ones. Where necessary, further information was sought from the corresponding authors of the studies by personal communication. Then, two reviewers compared and cross-checked the extracted data, and conflicts were adjudicated through consensus. We tested publication bias using Egger’s test.

### 2.5. Statistical Analysis

We separately calculated the cold and the hot estimate effects, as all of the studies included in our analysis did. The statistical analysis includes 2 stages: in the first stage, we converted all effect estimates into a relative risk; in the second stage, we obtained the ultimate effects using the meta-analyses.

#### 2.5.1. The First Stage

The first step of the stage is to convert ORs to RRs, using the following equation: RR=OR/[(1 – P0) + (P0 × OR)], where P0 = the incidence of non-exposed group. When the value of P0 is extremely small, we assume RR = OR. In all of our case-crossover studies, where ORs comes from, the incidence rate of stroke on a single day was consistent with a Poisson distribution, which we looked on as a small probability event. Therefore, P0 was considered extremely small. Therefore, RR = OR.

In some of the included studies, the authors provided U-, V-, or J-shaped curves, in which RR was calculated comparing a percentile to another percentile of the temperature. The second step of this stage for us was to provide estimates per an absolute change in this range. For most of the studies that provided the percentiles, for the cold effect, we reached the new RRs comparing the 1st to 25th percentiles of the temperatures; and for the hot effects, we used the 99th and 75th percentiles. We calculated log-RR divided by the range of the temperature percentiles in each specific area [17].

#### 2.5.2. The Second Stage

In the second stage, a meta-analysis was used to pool the estimates of RR from all the included cities. In other words, we pooled the city specific effect estimates. We calculated the *I^2^* statistic to evaluate the city-specific estimates. The meta-analyses were fitted using a random effects model if *I^2^* > 25%. Otherwise, we chose the fixed effects model.

Previous studies applied the Bayesian hierarchical models to pool the city-specific effect estimates and took latitude and lag patterns into consideration. But they also proved that latitude had little effect on respiratory and cardiovascular diseases [17].

Because the outcomes of the studies included varied widely, while each subgroup comprised relatively fewer studies, we defined the total outcome as MACBE, which include death (mortality due to stroke), occurrence of stroke (morbidity), emergency room visit for stroke (morbidity), and emergency hospital admission (morbidity).

For studies that only gave out stratified RRs instead of the overall ones, each subgroup was included. As we pooled the city-specific estimates, if a city had no pooled result, we pooled the subgroup effect estimates before the final total analysis. If the final analysis comprised two estimates from one city, we chose the one that was conducted in a larger population instead of smaller group in the city, such as a district in the city. If the outcomes in the two studies were different mortality or morbidity, we include both of them. If the outcomes and the study population were the same, we chose the one conducted at a later time.

As many studies provided different lag patterns of exposure to estimate the delayed effect of temperature change, such as single day lag, and cumulative lags, even the moving average of the previous 2 hours, when we pooled RR, we chose the largest estimate effects.

We also conducted sensitivity analyses to assess the stability of the results and investigated the heterogeneity. According to the subgroup analyses of most of the included studies, the sensitivity analyses were performed on the basis of outcomes (morbidity and mortality), types of stroke (ischemic stroke and hemorrhage stroke), age (≥65 and <65), gender (male and female), and lag patterns. Lag expression varied from the specific day of onset of stroke to an average of 28 days before event. We only pooled when more than three estimates were available (coming from 3 different articles). We did not compare the results due to different study designs, for those designed as the case-crossover studies were less than 3 passages in each group.

All the analyses were conducted by Microsoft Excel 2010 and Stata 12.0 (Stata Corporation, College Station, Texas, USA).

## 3. Results

**Figure 1:**
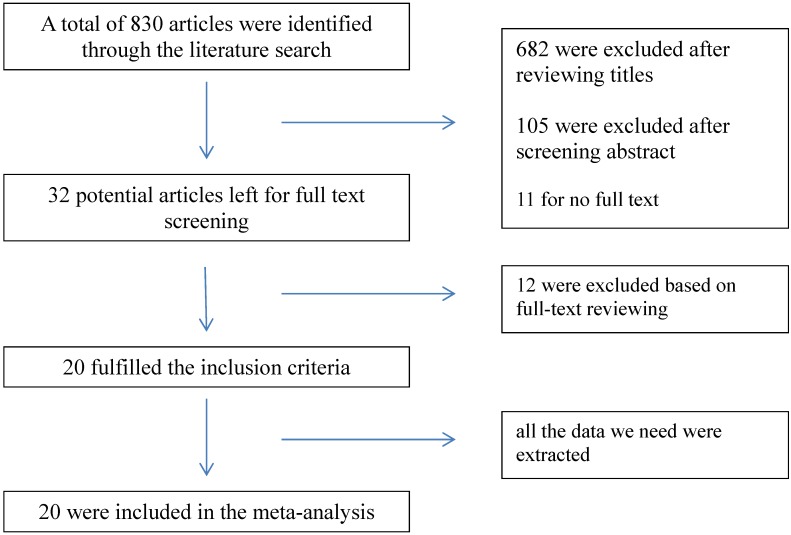
Flow chart of information through the different phases of a systematic review.

As is shown in Figure 1, 830 articles were included in the initial search. Of these, 682 were excluded after reviewing titles because they were absolutely not qualified for the inclusion criteria. Then, an abstract scanning was conducted and 105 were abandoned for they mentioned nothing relevant to our purpose in the abstract. Of the remaining 43 articles, 11 were deleted for not providing the full text of the articles, two provided estimate effects based on temperature range [18,19], four showed interests in cardiovascular diseases, while stroke did not appear as a subgroup [20,21,22,23], three only found a positive relationship (*p* ＜ 0.05) without an RR/OR [24,25,26], one focused on the daily temperature range [27] and stroke and two were searching for the long-term relationship [28,29].

The characteristics of the articles finally included are listed in Table 1. For the included 20 articles, the location of them varied widely from Asia to Oceania, mainly from nine countries. The retrospective periods differed from 1 to 19 years. Of these, 14 applied time-series design and the rest used case-crossover design. To control the variables, different models, such as generalized linear model (GLM), generalized additive models (GAM), generalized estimating equation (GEE), and distributed lag nonlinear model (DLNM), were constructed. Twelve used morbidity as the main outcome, seven and eigtht for hot and cold effects, respectively. Some studies explored both hot and cold effects. Seven studied the relationship between temperature change and mortality. Sensitivity analyses mainly included subtypes of stroke (HS and IS), age, gender, and lag patterns if possible. No obvious publication bias was found after testing, except the combined effect for MACBE in the cold days.

**Table 1:** Characteristics of the included studies.

Authors and year of publication	Outcomes Investigated	Published journal	Location and Period of Data Obtained	Study Design	model	No. Events	Variables Controlled	Lags
Kyobutungi *et al.* [30], 2005	stroke morbidity	Eur. J. Epidemiol.	Germany, 1998–2000	case-crossover	conditional logistic regression model	303	not mention	both
Wang *et al.* [31], 2014	emergency room visits	PLoS ONE	China, 2000–2009	time-series	DLNM	6,962	air pollution, pneumonia and influenza, holidays, DOW, trends	both
Dawson *et al.* [32]，2008	stroke admissions	Acta Neurol. Scand.	Scotland, 1990–2006	time-series	negative binomial regression	6,389	trends, season and DOW	null
Chen *et al.* [33], 2013	stroke mortality	Neurology	China, 1996–2002	time-series	GAM and DLNM	127,750	risk factors, socio-demographic characteristics, air pollution and relative humidity	both
Matsumoto *et al*. [34], 2010	stroke morbidity	J. Epidemiol.	Japan, 1995–2005	case-crossover	Multilevel logistic regression	450	age, obesity, smoking, total cholesterol, systolicblood pressure, diabetes, all the meteorologicalparameters	null
Hori *et al.* [35], 2012	emergency admission	Int. J. Environ. Health Res.	Japan, 2006–2010	time-series	generalized linear Poisson regression model	778	DOW, holidays, influenza, air pollution, other meteorological factors	average
Morabito *et al.* [36], 2011	stroke admissions	Stroke	Italy, 1997–2007	time-series	GLM	112,870	temporal variables, categorical factors	single day
Hong *et al.*[37], 2003	stroke admissions	Epidemiology	Korea, 1998–2000	case-crossover	conditional logistic regression model	545	humidity and air pressure	single day
Wang *et al.*[38], 2013	hospital admissions for ischemic stroke	PLoS ONE	China, 1990–2009	time-series	DLNM	1,908	season, trend, DOW and public holidays, season	both
Atsumi *et al.* [39], 2013	cardiovascular mortality	Circ. J.	Japan, 1993–2008	time stratified case-crossover	conditional logistic regression model	1,709	relative humidity and air pollution.	both
Mostofsky *et al.* [40], 2014	stroke morbidity	Cerebrovasc. Dis. Extra	USA, 1999–2008	Time stratified case-crossover	conditional logistic regression model	1,763	PM 2.5, ozone and relative humidity	both
Breitner *et al.* [41], 2014	cardiovascular mortality	Heart	Germany, 1990–2006	time-series	DLNM	187,943	trend, season, DOW, influenza, relative humidity and barometric pressure	both
Wang *et al.* [42], 2009	stroke admissions	Int. J. Biometeorol.	Australia, 1996–2005	time-series	GEE	12,387	humidity, PM10,NO2, O3 and SO2	null
Cevik *et al.* [43], 2014	Emergency stroke admissions	Int. J. Biometeorol.	Turkey, 2009–2010	time-series	GAM（generalized additive models） and DLNM	373	wind speed and air pressure	single day
Basu *et al.*[44], 2012	emergency room visits	Epidemiology	USA, 2005–2008	time-series	conditional logistic regression model	1,215,023	air pollution	both
Green *et al.* [45], 2010	hospital admissions	Int. J. Public Health	USA, 1995–2005	case-crossover	conditional logistic regression model	91,806	season，DOW，air pollution	both
Zhang *et al.* [46], 2014	cerebrovascular mortality	Environ. Health	China, 2004–2008	time-series	DLNM（distributed lag nonlinear model）	20,308	season, trends，DOW, relative humidity, air pollution	average
Shaposhnikov *et al*. [47], 2014	hospitalizations	Int. J. Biometeorol.	Russia, 1992–2005	time-series	generalized linear Poisson regression model	1,096	DOW, and geomagnetic storms	single day
Lim *et al.* [48], 2013	stroke mortality	Int. J. Biometeorol.	Korea, 1992–2007	time-series	GAM GLM	149,598	humidity, air pressure, air pollution, DOW, season, and year	both
Goggins *et al.* [49], 2012	stroke admissions	Int. J. Biometeorol.	China, 1999–2006	time-series	GAM	130,962	DOW and holiday, air pollution, other meteorological factors, influenza rates, season and trends	both

Arranged by the titles of the articles, not listed.

### 3.1. Overall Analyses

**Figure 2:**
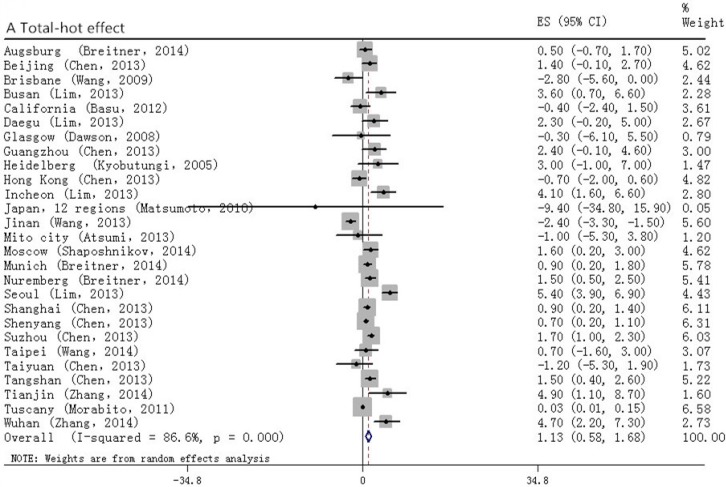

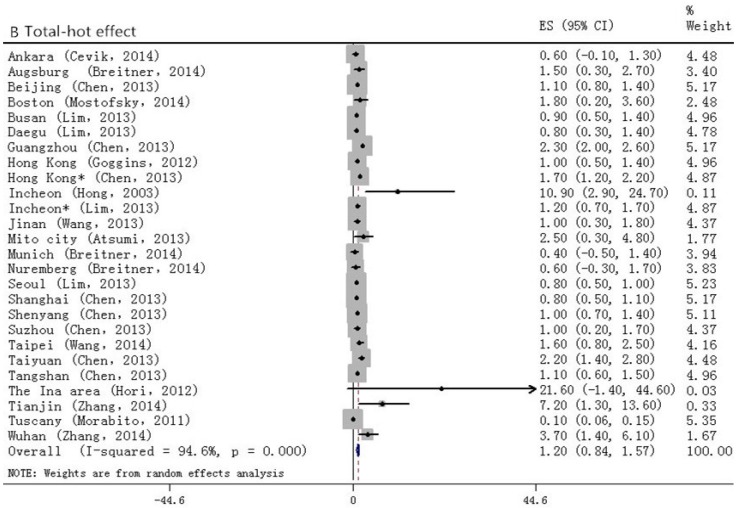
Forrest plots for total analyses: relationship between the temperature change and the onset of MACBE. A and B stand for hot and cold effect, respectively.

The overall pooled results (Figure 2) showed that for the hot effect, 1 °C increase in the temperature brought a 1.1% increase in the occurrence of MACBE; and for the cold effect, a 1 °C decrease in temperature associated with 1.2% increase in MACBE. Furthermore, we analyzed the health effects, morbidity and mortality, separately.

**Figure 3:**
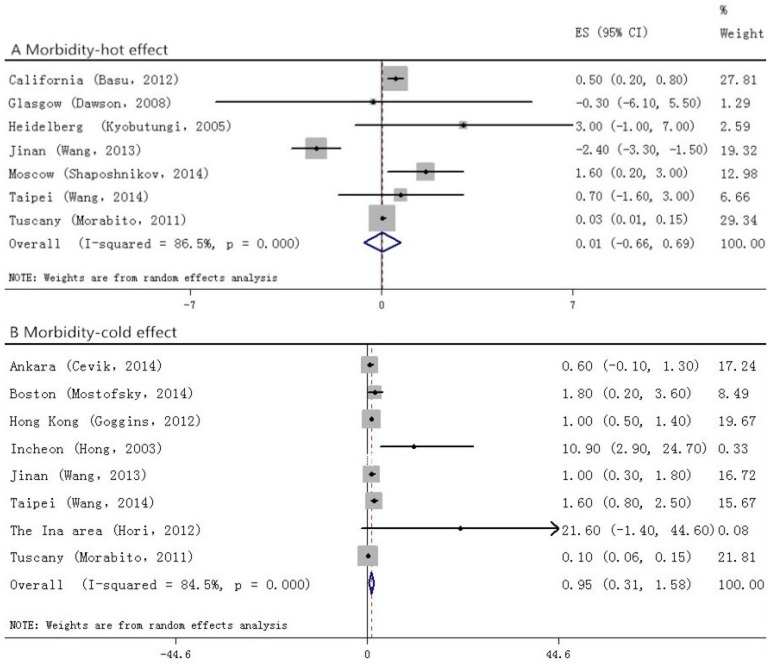
Forrest plots for relationship between the temperature change and stroke morbidity. A and B stand for hot and cold effect, respectively.

**Figure 4:**
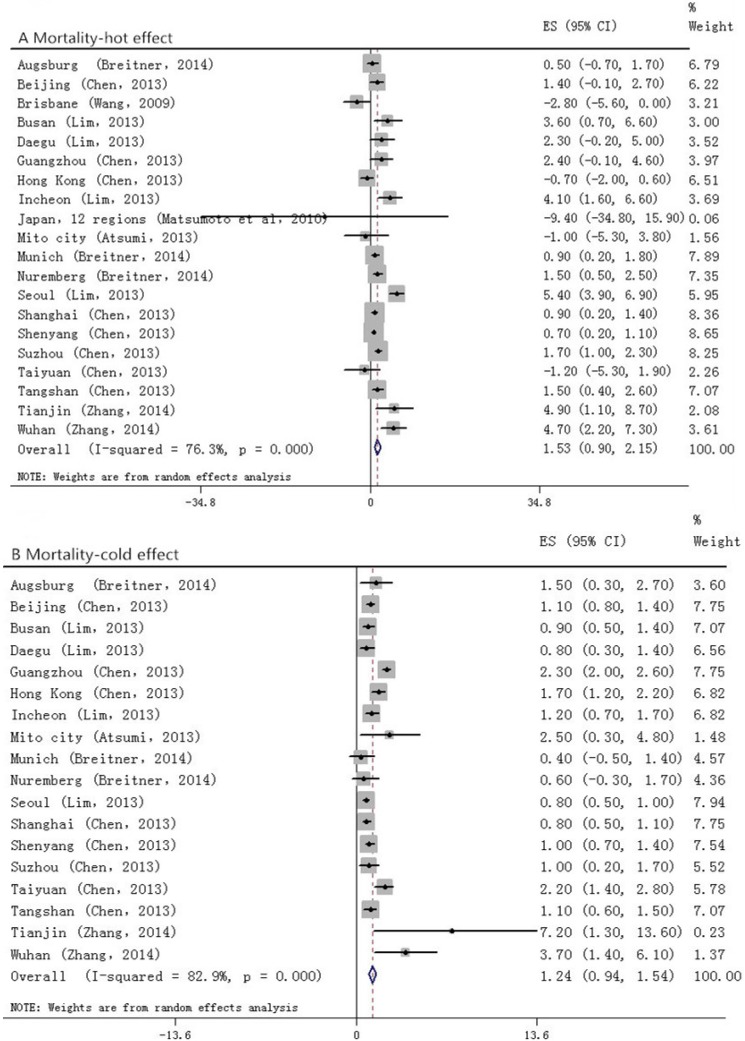
Forrest plots for relationship between the temperature change and stroke mortality. A and B stand for hot and cold effect, respectively.

**Figure 5:**
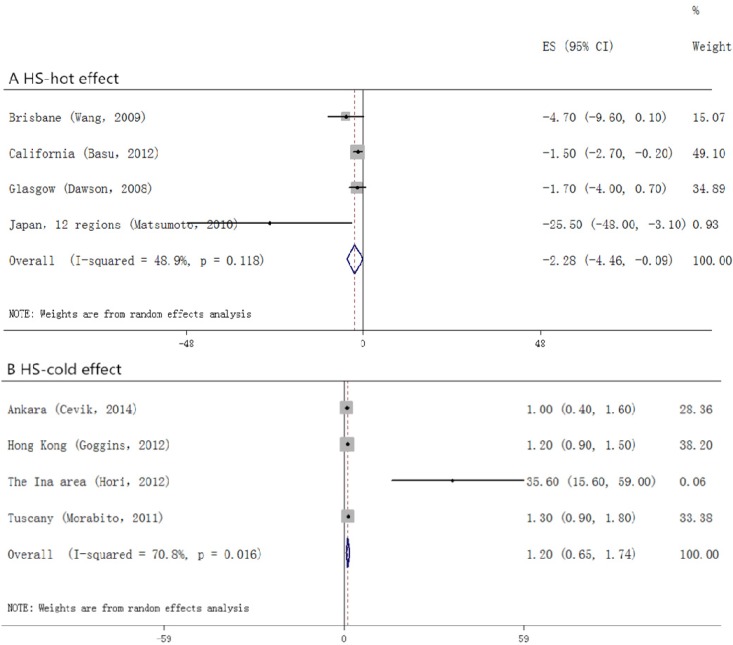
Forrest plots for relationship between the temperature change and HS. A and B stand for hot and cold effect, respectively.

**Figure 6:**
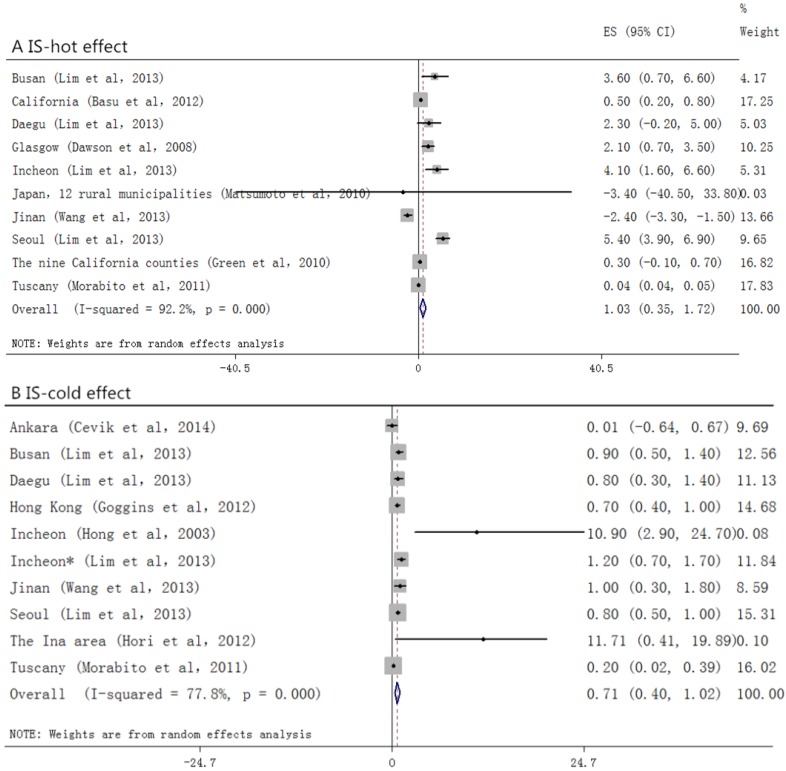
Forrest plots for relationship between the temperature change and IS. A and B stand for hot and cold effect, respectively.

Among the studies that took morbidity as the outcome (Figure 3), we did not find positive relationship between temperature change and stroke (0.0, (−0.7 to 0.7)) in the hot effect, while in the cold one, a 0.9% (0.3 to 1.6) increase had been seen. For those who suffered from stroke and died (Figure 4), a stronger association had been found. In the hot effect, 1 °C increase in temperature related to 1.5% (0.9 to 2.2) increase in death, while in the cold, the percent change appeared to be 1.2% (0.9 to 1.5). As several of the included studies took only IS or HS as the outcome variables, we also analyzed each of them. In the studies observing HS (Figure 5), the hot temperature appeared as a protective factor, a 1 °C increase in temperature related to −1.9% (−2.8 to −0.9), by contrast, the cold temperature acted as a risk factor. Temperature changes acted as risk factors in both hot and cold effects for IS (Figure 6). *p* values for Egger’s test for overall analysis can be found in Table 2.

**Table 2:** *p* values for Egger’s test in overall analyses.

Egger’s test, P	Total	Morbidity	Mortality	HS	IS
Hot effect	0.196	1.000	0.536	0.221	0.803
Cold effect	0.071	0.711	0.148	0.734	0.178

* Mortality for the same city.

* Mortality for the same city

### 3.2. Sensitivity Analyses

To test the stability of the overall analyses, we conducted sensitivity analyses according to age, gender, and lag patterns, where there are no less than three articles in each group. Details can be seen in Table 3, Table 4 and Table 5.

For those who were 18 to 64 years old, temperature appeared to be an irrelevant factor. However, for the elderly (65 years or older), the relationships were stronger. The percent change for the hot days and the cold days were 0.8% and 0.5%, respectively.

For males in the hot weather, a 1.7% increase is seen and 0.7% for cold days. Among females, we found negative relation in the warm days, but a positive one for cold days.

Due to the various lag patterns among the under-analyzing studies, we only pooled lag0 and lag1, which means the temperature measured on the same day or previous day MACBE occurred, for hot effect and lag0, lag1, lag2, lag3, lag4, and lag02 (moving average for the past 72 hours) for the cold effect. In the hot days, we found a strong exposure–response relationship on lag0, whereas no significant relationship for lag1. In the cold days, the effects were different. Stronger associations were found on lag2, lag3, and lag4. The moving average for the previous 72 hours (lag02) also showed an significant result 1.0% (0.0 to 2.1).

**Table 3:** Sensitivity Analyses for Hot Effects.

Subtypes	<65	≥65	Male	Female	Lag0	Lag1
Number of estimated areas	7	7	6	6	6	3
Number of estimated articles	4	4	3	3	4	3
Effect Size (95% CI)	1.000 (1.000–1.001)	1.008 (1.002–1.015)	1.017 (1.003–1.030)	1.019 (0.993–1.046)	1.045 (1.007–1.082)	1.010 (1.000–1.019)
Heterogeneity, *I^2^,%*	0	78.4	0	62.3	93.2	9.9
Publication Bias (Egger’s test, *p*)	0.368	0.260	0.260	0.707	0.216	0.540
Model	Fixed	Random	Fixed	Random	Random	Fixed

**Table 4:** Sensitivity Analyses for Cold Effects: Age and Gender.

Age and Gender	<65	≥65	Male	Female
Number of estimated areas	6	6	5	5
Number of estimated articles	3	3	3	3
Effect Size (95% CI)	1.001 (1.000–1.002)	1.005 (1.001–1.009)	1.007 (1.002–1.011)	1.009 (1.004–1.014)
Heterogeneity, *I^2^*,*%*	0	60.9	40.4	51.8
Publication Bias (Egger’s test, P)	0.133	0.133	0.452	0.566
Model	Fixed	Random	Random	Random

**Table 5:** Sensitivity Analyses for Cold Effects: Lag patterns.

Subtypes	Lag0	Lag1	Lag2	Lag3	Lag4	Lag02
Number of estimated areas	9	4	11	4	11	7
Number of estimated articles	6	4	4	4	4	3
Effect Size (95% CI)	0.999 (0.997–1.002)	1.006 (0.996–1.016)	1.003 (1.001–1.004)	1.007 (1.002–1.012)	1.002 (1.001–1.003)	1.010 (1.000–1.021)
Heterogeneity, *I^2^,%*	41.4%	27.6	50.9	0	40.8	38.3
Publication Bias (Egger’s test, P)	0.002	0.308	0.349	0.734	0.814	0.764
Model	Random	Random	Random	Fixed	Random	Random

## 4. Discussion

To our best knowledge, this study is the first-ever meta-analysis to investigate the association between temperature and stroke. We came to the conclusion that cold temperatures had a positive relationship with both mortality and morbidity of stroke, and the relationships could also be seen for the mortality in the hot days. Hot days may act as a protective factor in hemorrhage strokes. The sensitivity analyses were consistent with the results from the main analyses, and there were no published bias in the analyses. Thus, the results were reliable. 

Stroke is listed as the second leading cause of death from GBD 2010 [10] and caused about 10% of the 52,769,700 deaths worldwide in 2010 [10]. Our study found a positive relationship between cold temperatures and health effect such as mortality and morbidity of stroke. While we found positive links between hot temperature and MACBE, no apparent associations were found between temperature change and morbidity. The potential biological mechanisms have not been well-understood. The winter months were associated with a number of adverse physiological parameters, such as serum lipid (especially cholesterol), blood glucose and serum fibrinogen concentration. Fibrinogen was looked on as a predictor of both ischemic heart disease and stroke events [50]. Low temperature also led to the vasoconstriction of the peripheral blood vassals. Acute cold exposure has been demonstrated to cause platelet increasing and hemoconcentration [51]. In terms of all the potential mechanisms listed above, the cardiovascular and cerebrovascular systems turned to be inactivated in winter, leading to the occurrence of such events. Higher temperatures were also associated with hemoconcentration and the impairment of peripheral vascular endothelial function [52].

We noticed in our review that high temperature had little effect with stroke morbidity. Previous studies showed a complex relationship. For example, a study in Taiwan compared stroke admission between warmer days (≥20 °C) and cooler days (<20 °C) and demonstrated increased risk of stroke admission for both HS and IS [20]. While a few studies support our result, Nitschke *et al.* analyzed the effects of all heat waves affecting Adelaide between 1993 and 2006, and found no significant change in the rate of stroke admission [53]. Similarly, a 2013 investigation carried out in Sydney between the years of 1991 and 2009 has also found no increase in stroke admissions [54]. Dawson *et al.* [32] also found a non-significant association (*p* = 0.17). One possible reason for the inconsistent outcomes was that the studied cities were in different climate zones. We covered eight areas in our analyses, coming from sub-tropical climates to temperate climates. Those who live near the equator have adapted to the hot temperatures, both physically and biologically. Another possible reason was that most of the places under evaluation were from the developed countries or the capital and economic center of the developing ones. During heat waves, they may stay at home instead of conducting outdoor activities, which could affect the results. Moreover, the candidates for stroke may die instead of making an emergency room visit, which might also partly explain the negative finding [55].

Further exploration showed that higher temperature acted as a protective factor on HS, whereas a risk factor for IS. This may be different from other studies [35,56]. A possible reason for this discrepancy might be differences in the climate, population characteristics, and proportion of stroke subtypes of the studied areas. When analyzing the subtypes of stroke, we combine the outcomes of both morbidity and mortality. As the number of articles was limited, we cannot separately pool the estimates. This phenomenon can be explained as, in hot weather, the peripheral vessels relax, lowering the afterload and reducing blood pressure, thus preventing the ongoing of HS. However, as heat continues, surface blood circulates faster, and more water comes out to accelerate the process of heat elimination. As excessive water evaporates, hemoconcentration and electrolyte imbalance appears [57,58,59,60].

The results from the sensitivity analyses implied that males were vulnerable to stroke in the hot weather. However, on cold days, the effects for both sexes appeared to be larger, and especially for females. More explorations have been conducted to confirm these results [61,62,63]. The reason might come from estrogen, which could enhance the activity of adrenergic alpha2C-receptor, leading to the vasoconstriction in both superficial and deep arteries [62,63,64,65]. In addition, we found the same conclusions as many other studies—the old were more sensitive to the change of temperatures. The slower reaction of the regulation system makes them more vulnerable to temperature effects [66,67].

Many studies have reported a relatively shorter period of hot effect than for the cold one. The cold harmed the health and could last for a relatively long time—two weeks or more—while heat waves came and went quickly [8]. Although the pooled effects of lag effect were influenced by the number of articles, we could observe the trends. For those not listed in the combined estimates, many supported the idea [33,38,39,40,41].

Risk-stratified analyses from the included papers showed that those who had a BMI < 25, or developed hyperglycemia might have a better chance of being influenced by cold temperature, whereas smoking status, alcohol intake and hypertension contributed little [37,39]. Only one study compared the effects among different races—white, black, Asian, and Hispanics [44]. It looked on Hispanics as the susceptible group, which suggested a higher possibility of being affected by temperature. As large amount of studies have been conducted on Asian and Caucasian ethnicities, more are needed in various races.

Three of the studies in our analyses used the apparent temperature (AT) as the exposure factor, which was calculated using the method as described by Steadman in the norms of AT [68], which can be calculated using air temperature (°C), water vapor pressure or humidity (hPa), and wind speed (m/s), and relative humidity (%). Combining the four meteorological factors together, to some extent, represents the whole climate. If more research applied AT, we would carry out a comparison between the traditional ambient temperature and AT. Daily temperature range was another measure for the exposure–response relationship, but few of those studies could be seen. In the following work of our task force, we would take all the measures into consideration.

Heterogeneity may be related to inherent differences between the studies, as well as their design and statistical analysis. The study areas and population may play an important role. Different areas located in different meteorological zones, the average temperature, relative humidity, wind speed, wind directions, and air pressure were different. Although air pollutants, considered as confounders in most of the studies, were introduced to control for their confounding effects, they were considered in different ways. Covariates included in analyses differed, and different models used for analyzing might also be contributors, thus leading to heterogeneity in study results. The limited number of articles stopped us from comparing the designs of studies, however, difference between time-series design and case-crossover design for the pooled risk estimation between exposure–response relationships has been found elsewhere [69]. Other factors, including study period, the risk factors for the studied population, economic conditions, may take part as well.

Publication bias occurs when the publication of research results depends not just on the quality of the research but also on the hypothesis tested, and the significance and direction of effects detected [70]. In our work, publication bias was found in lag0. As we can see in Figure 1 and Table 4, we include 13 articles (26 places) in the cold effects, whereas only six of them give the RRs in lag0. The papers already published still have not listed the RRs in lag0, not to mention the unpublished work. We found negative association in lag0 with publication bias. So we guess it will still be negative without the bias.

Notable points in this work must be considered. First, even if we concluded all the published articles in this systematic review, the limited number of studies and places stopped us from stratifying studies by more confounding factors. As most of the studies are from the capital cities in developed and developing countries, the results might not reflect the conditions of the whole world. Second, when pooling the estimates, we chose the largest effects for each study, which might exaggerate the real situation, although we did take different confounders into consideration in our sensitivity analyses to test the stability of the results to ensure the results were not distorted. We just want to give out the largest possible estimates to arouse attention from the public to protect themselves from the changeable environment. Third, we concentrated on the influence of temperature and stroke; however, different measures such as maximum temperature, minimum temperature, and AT were not analyzed separately. Further measures of DTR and other influence of meteorological factors for stroke and other ending points such as cardiovascular diseases and respiratory diseases are to be considered.

## 5. Conclusions

Our study suggested that both an increase and a decrease in temperature had a marked relationship with the occurrence of MACBE. In terms of the ending points for stroke such as morbidity, mortality, HS, and IS, temperature change might play a role.

## Abbreviations

CNKI: China National Knowledge Infrastructure
MACBE: major adverse cerebrovascular events
CI: confidence intervals
HS: hemorrhage stroke
IS: ischemic stroke
NE: note express
GBD: global burden of diseases
DOW: day of the week
RR: relative risk
OR: odds ratio
EN: expected number
AF: attribute fraction
GLM: generalized linear model
GAM: generalized additive models
GEE: generalized estimating equation
DLNM: distributed lag nonlinear model
AT: apparent temperature
